# Sociology and The Complexity of What Is Missing

**DOI:** 10.1111/1468-4446.70077

**Published:** 2026-01-24

**Authors:** Konstantinos Poulis

**Affiliations:** ^1^ Middlesex University London UK

**Keywords:** complexity, entities, epistemology, missing, ontology, presence

## Abstract

What is ‘missed’ by sociological literature underpinned by assumptions of presence that a *missing* approach can rectify? I appropriate a metaphysics of presence and an alternative focus on what is missing as ontological foci to revisit complexity studies in sociology. I review key themes therein and show that, by predominantly adopting a being‐laden set of metaphysical assumptions, the complexity discourse overlooks subtler and more nuanced aspects of elucidating social settings. By attuning ourselves to what is missing, I make a case for what the possible consequences of this overlooking might be while showing the theorizing inadequacies of complexity thinking, which rests squarely on tangibility and observability of Aristotelian entities.

## 1 Introduction

Inquiry into the social is punctuated by entitative dualisms: actors and institutions; agency and structure; collectives and individuals. Such bifurcations demarcate the reality we attend to and investigate and are permeated by a characteristic: They are predicated upon something substantial and actual that is analytically circumscribed as ‘entity/ies’. A state or a region; a community; technology, gender groups, race, class are parsed and classified as entities in sociological conceptualizations (Wajcman 2008; Hay 2014; Seehuus 2019). Entities are omnipresent empirical units while being depicted as discrete variables in modelling attempts and representational designs. Furthermore, their contingent linkages represent the way we understand ‘complexity’ as a ubiquitous notion in sociology (Castellani and Gerrits 2024; Page 2015; McLennan 2003; Stehr and Grundmann 2001). Given their centrality in our discourse, I use complexity as a case in point to problematize the ontological reification of entities as exclusive building blocks of theorising. Namely, this exclusivity bias among complexity scholars creates an illusion: Social reality and its complexity are all about *presence and beings*.

Problematisation of this kind does not imply anything ‘wrong’ about entities per se nor do I seek to negate the value of entitative commitments in sociology. After all, entitisation of reality accords with sociologists' focus on the observed, the seen, and the manifest. It reflects an understandable tendency in much of the sociological literature to treat reality as a ‘thing’ (see Scott 2018) and the anthropomorphic tendency to reify phenomena (e.g., institutions and their logic) as if they are real in a materialistic sense (so‐called *organicism*; see Castellani and Hafferty 2009). Then, the appropriation of entities by sociologists is rather expected, it has offered exceptional results and, reasonably, entities constitute the backbone of theorising in our field.

However, a *mono‐dimensional* appropriation of entities within complexity studies in sociology is paradoxical. Namely, what is *not* present (e.g., silence, loss, absences, gaps, voids, mortality, lack, omissions) is characterized by a phenomenological incongruity between expectations and experiential reality that induces strangeness, unfamiliarity, and unsettling oddness (Cavedon‐Taylor 2017; Farennikova 2013). What could be more complex than such states? While this incongruity has been elaborated in general sociology (see Scott 2018; Poulis 2025), *complexity studies* therein neglect such advances.

For example, emergence ‐a core complexity concept‐is the central puzzle of what we are, where we come from and where we are heading (Padgett and Powell 2012). To explain emergence, scholars' focus is on tangible cues such as regional networks or political ties and on manifest actors such as economic elites and high‐tech clusters. We appropriate such ‘tangibles’ to explain the seeds of emergence or complexity. Yet, these also come about through reflection upon what is missing that is, *because of* it. The unavoidable mortality of ours may paralyse; the impoverished state of a community may catalyse restorative action; the absence of a leader may undermine social movements. Sociological scholarship recognises this fecundity of missing‐ness. Yet, the complexity discourse, by emphasising ‘tangible’ cues and ‘present’ circumstances, downplays how agents enact or foresee new possibilities through missing‐ness.

So, while agentic traditions are evident in complexity‐informed sociology, this paper foregrounds agency and the reflexive capacities of social actors through a missing lens. Via such a lens, I aspire to add to the literature by articulating missing elements as distinct empirical possibilities and as cues of moral, symbolic or practical significance. Missing a relative during a family gathering or losing an object that is dear since childhood infuses meaning, yields emotions or ignites reflective deliberation (Holmes and Ehgartner 2021). Evoking missing‐ness signposts that something may be ‘wrong’, too. Yet, associated memories also galvanize us to restore order and create a new self or enact a new environment (Poulis 2025). Either way, ignoring the constitutive role of missing‐ness is a substandard representation of how social entities implicate reality and, thus, a suboptimal account of complex dynamics in social settings.

So, this study problematises a neglect that is, the role that missing‐ness plays in complexity arrangements. This neglect is an oxymoron for two reasons. First, complexity scholars claim a comprehensive understanding of social contexts due to their focus on multiple interactions among entities (Page 2015). However, how comprehensive can this understanding be if it denies the other half of what sociologists seek to disentangle? Second, the macro‐view that emerges from those micro interactions is largely the raison d'être of complexity scholarship. Yet, if scholars ignore micro nuances that matter, how accurate or helpful are complexity studies in sociology? After all, their promise has been to provide a panoptic view of how social structures evolve and behave (Page 2015).

Then, current forms of theorizing may limit understanding of complexity. Namely, approaching complexity via ‘beings’ acts as a testimony that some sociologists have inherited a legacy, which commits to an entitative view of reality and a natural focus on presence. Instead, this study contends that *complexity may be also brought about by something* i.e. *missing.* Then, seeing missing elements as ephemeral, less important deviances reduces the potential of complexity to be relevant for sociology. So, can we refine substantialist limits through missing‐ness? Would complexity studies benefit from such a lens? I contend ‘yes’ and structure the remainder as follows. First, I conceptualize ‘missing‐ness’ and stress some caveats. Then, I discuss the link between sociology and complexity, with reference to sociology of organizations as an archetypical sub‐field that exemplifies requisite arguments. I go on to demonstrate entitative commitments of extant scholarship and propose a parallel treatment of missing‐ness when theorizing complexity in social settings. I conclude by discussing onto‐epistemological concerns.

## 2 Conceptualizing Missing‐Ness

Ever since Gorgias, philosophy offers fascinating accounts of how we experience, perceive, or feel manifestations of non‐beings, gaps, loss, omissions, almost nothings, or non‐existents (e.g., phenomenological or metacognitive accounts; Cavedon‐Taylor 2017; Gow 2021; Farennikova 2013). Out of this body, I appropriate missing‐ness in role functionalist terms (Tiehen 2015) and understand it as *the perceived missing status of an entity that could be otherwise expected in social realms* (e.g., a state resource, a communal procedure, a material asset). Importantly, I am interested in this kind of missing‐ness since our perception of it can catalyse events in social settings, including organizational ones (see Scott 2018; Poulis 2025).

First, this kind has a tactility that is important for decision‐making. In organisations, decisions are key and missing a causal entity is a property that leads to profound outcomes (Dowe 2009). Second, this kind is cognitively accessible via comparison; a process that is laden on the enduring representation of this entity in one's memory and cognition and juxtaposed with perceptual input from their surroundings, which signals that this entity is missing (Barton 2020). This comparison becomes agentic and calls actors to confront lack, gaps, absence, silence as instantiations that generate adaptive responses, induce meaning and enact new practices (Page 2015; Poulis 2025).

Nevertheless, our Aristotelian empiricism prevails with complexity scholars assigning ontological priority to beings. Notwithstanding the value of doing so, it is also prudent to recognize another perspective: What is missing in social settings is neither semantically neutral nor merely a negation of presence; it may be deeply consequential and, hence, constitutive of complexity arrangements. For example, human agents are not only constituted through, within and by entities for example, the institutions they inhabit (the typical framing in institutional theories). They are also defined via lack of institutions or their extinction (e.g., see how institutional voids give shape to remedial action; Mair et al. 2012). Thus, if order or emergence can be shaped via entities *and* via what was, is or may be missing, too, then, complexity scholarship cannot neglect the constitutive role that missing‐ness may play in inducing complex regimes.

### 2.1 Setting the Scene

The audience of interest here is sociologists employing complexity theories and complexity theorists studying social facets of complexity. I do not exclude others e.g., cyberneticians who study natural settings. However, different commitments prevail in non‐social realms. e.g., complexity is used by non‐Euclidean geometry scholars to measure coastlines as fractals. However, the epistemic toolkits and the scholarly orientation therein are different. Notwithstanding their value, fractals may be less relevant to social inquiry whereas sociologically relevant approaches (e.g., second‐order cybernetics) may not suffice to address concerns in non‐social domains. Then, I stress some caveats as organizing structures for the study.

First, an Aristotelian privileging of entities is not discarded. Hence, the purpose is not to condemn sociological scholarship for its insistence on what ‘there is’. Rather, I seek to push back and claim that, in social settings, there is another driver of complexity, too that is, elements of missingness such as voids, silences and non‐events. Such a parallel recognition unsettles our categories and demands to be theorized in a more nuanced way. So, as shown below, by shifting ontology to the big unknown; epistemology to articulation over measurement; and analysis to the possibility of inaction as much as adaptation, I seek to reframe complexity as a field constituted as much by absence as by presence.

Second, this reframing calls us to incorporate non‐events, missing data or withheld information in our accounts of systemic emergence and environmental dynamism. This cannot be done via a mechanistic Newtonian treatment and the same itemized and measurement‐linked tools we are accustomed to. This does not imply that alternative modes are not found in sociology. For example, studies rooted in pragmatism such as actor‐network theories (Lee 2024), ignorance studies (McGoey 2012), or studies on that which is rendered ‘invisible’ (e.g., invisible work; Star 2024) are about standpoints from where it is possible to shine a light on something, which is absent from the purview of orthodox epistemologies. Moreover, the whole pragmatic tradition of abductive inquiry (see Swedberg 2016) or critical feminist epistemologies (e.g., Fleetwood 2019) may be seen as forms of knowledge‐creation predicated on missing‐ness. However, such streams are not strictly about complexity but fine albeit non‐complexity examples that deviate from the entitative mainstream.

Third, herein, complexity is treated as a scholarly discourse. However, an academic approach often fails to recognise that complexity is linked to a set of practices embedded in policy and applied problem‐solving. Then, some of the tensions identified below arise from how complexity is operationalised under institutional pressures, rather than from theory alone e.g., libertarianism in Santa Fe traditions may stem from institutional pressures for funding purposes, not from a sense of theorical purity.

Fourth, the paper may problematise what complexity scholarship does or fails to do, but the critique herein does not seek to attack entire complexity traditions. e.g., one may distinguish between social physics and Santa Fe–style approaches with several computational scientists doing social inquiry. Therein, social science traditions of simulation (e.g., Gilbert 2007) have long been process‐oriented and concerned with emergence and macro–micro relations. Therefore, seemingly uniform strands are, in fact, not part of a single, object‐focused tradition. Rather, healthy pluralism is spotted even within tightly demarcated fields. Therefore, my critique of object‐laden formalisation should not be seen as a general scepticism towards modelling but as a targeted critique of what complexity modelling cannot or is unwilling to capture in relation to missing‐ness. Hence, the issue is scope and limits, not legitimacy of any extant attempt.

Fifth, sociology is *not* only about entities. For example, wide parts of sociology subscribe to a process approach and note that ‘both agency and structure are emergent properties of social interactions/relations’ (Crossley 2022, 166). Yet, why can't there be a parallel emphasis on relationships that never occurred, links that will never materialise or bonds that ceased to exist? e.g., in public bureaucracies or social collectives, a spatialized focus on entities (e.g., resources, departments) and their linkages (collaborative schemes, networks etc.) is used to explain the success of organizational arrangements. Yet, there is a disproportionate emphasis on non‐interconnections (e.g., relationships that should have been but are not). This lacuna is crucial since a unilateral emphasis on what ‘there is’ omits critical cases of lack and, hence, presents a superficial view of causal dynamics. For example, failure may be better explained via lack of coordination rather than presence of resources. So, can complexity studies ignore missing‐ness?

## 3 Complexity in Sociology

For 50 years, complexity thinking ‘remained always unknown in physics, in biology, in social sciences’ (Morin 2007, 5) whereas, later, it was largely denounced by science as ‘superficial or illusory’ (Morin 2007, 2). Still, we see complexity concepts, which have been ‘*en vogue* for some time and then disappeared again’ (e.g., edge of order; Castellani and Gerrits 2024, 354). However, as the field grew (especially following Ashby's law of requisite variety; Poulis and Poulis 2016; Morin 2007), complexity elucidated not only impeccable order (e.g., ant colonies) or macro‐level determinism in natural realms (e.g., planetary movements) but also dispersion and dissolution in social settings (M. Williams 2021; Byrne and Callaghan 2022).

This complexity ‘turn’ in the 1990s enabled social sciences to move away from complexity as being synonymous with randomness or chance (Gerrits 2012; Urry 2005). This is exemplified in the many complexity themes that have been produced (e.g., chaos theory, dissipative structures, far‐from‐equilibrium; Page 2015; Burnes 2005) and the plethora of scholarly traditions such as evolutionism, computational sociology, the British school of complexity, general systems theory, Luhmann's legacy, postmodern, postcolonial and critical streams or methods linked to comparative or intersectional tools (see Castellani and Gerrits 2024 for a comprehensive overview). This turn also provided the theoretical scaffolding for core theories in our field (e.g., emergence, adaptive systems, configurational approaches, theories of order; Castellani and Gerrits 2024; M. Williams 2021; Castellani and Hafferty 2009) and shed new light on our main object (i.e., societies) via themes such as disorder, chaos, confrontation, exclusion, small‐world networks, social advantage, mobile hybrids, and triggers of social change (Lee 2024; Crossley 2008; Urry 2010; Room 2016; McLennan 2003).

At this stage, I ought to note: This historical evolution of the field from early cybernetics to modern socially grounded approaches was uneven and plural from the outset, rather than a smooth progression. So, the term ‘evolution’ does not imply that early complexity work was uniformly focussed on clearly bounded objects and actors. Rather, relational and process‐oriented ideas were also present early on, even if they were not dominant. So, any historical sequencing does not imply linearity or mere accumulation but a plurality that has always been replete of variegated traditions and contradictions as well as consensus and cross‐fertilisation. So, some of the implied or explicit critique herein is linked to which strands historically took hold rather than what existed at all.

Followjng this plurality, we now see a burgeoning literature, which connects sociological concerns (e.g., social mobility, economic development, inequality, global warming, job structuring) with complexity (Castellani and Gerrits 2024; Byrne and Callaghan 2022). The purpose is noble that is, the governance of societal challenges linked to health, social care, equality, or ecological disasters (Gerrits 2012; Room 2016; M. Williams 2021). Such streams are important in their own right. However, for this paper, they are important because they emphasize the *role of agency* in effecting change i.e., complexity is not only about mapping non‐linearity and meta‐structures, but also about the perceptual powers and will of those inhabiting complex regimes.

Of course, this complexity turn did not materialize without critique. Early on, some of those foci were seen as deviance from the ‘norm’ or as statistical irregularities. In the scientific canon where regularity, predictability and invariant laws prevail, such contingencies could not be part of mainstream sociological analysis. Rather, they were treated as empirical ‘noises’ or ‘nuisances’ that fell outside a Newtonian ideal. As such, several complexity themes have been left out of empirical analyses as residual epiphenomena, mere metaphors or ill‐defined buzzwords (e.g., edge of chaos or temporal states of criticality; Poulis 2021).

Additionally, we see disagreements about the ontological strata (Yang 2024) or epistemological commitments (M. Williams 2021) that scholars ought to appropriate. For example, the complexity promulgated by the Santa Fe Institute stands in contrast to European traditions of reflexivity. The former is grounded in a libertarian ontology and seeks definite mappings of complexity via predictive toolkits; the latter builds on complexity participants to surmise how complex phenomena are perceived to be (Baker 2022; Li 2021). This example is one important divide among several, alongside differences over for example, case‐based versus variable‐based work and policy‐oriented versus abstract modelling approaches.

Another example is configurational complexity, which refers to ‘a rigorous set of theories and methods for modelling social complexity’ (Castellani and Gerrits 2024, 254). It elucidates *cases* of complexity (replete with their biases, blind spots and prejudices) and sheds light on real‐life issues such as ‘democratic government or an effective school system’ (Castellani and Gerrits 2024, 256). However, divergent views are also observed (e.g., anti‐categorical, intra‐categorical, inter‐categorical complexity) in relation to systemic elements beyond the focal configuration (e.g., about trajectories).

Moreover, restrictive versus generalized complexity underlines a disconnect, with the former rejecting the latter as mere chattering and the latter critiquing the former as decomplexifying complexity while being ‘certainly inadequate to properly describe the social’ (A. Williams 2020, 30; Morin 2007). This is due to the open ontology of social spaces, which are premised on the *changeability* of constituent elements, not on their fixity as largely happens in natural systems (Gerrits 2012). This makes social challenges (e.g., public policy ones) ‘much harder to solve’ since ‘there is no consensus about the nature of the problem and the nature of the possible solution’ (Castellani and Gerrits 2024, 352). Thus, complexity is not a monolith but an evolving field where variegated traditions co‐exist (Byrne and Callaghan 2022; Castellani and Hafferty 2009).

Notwithstanding such instances of disconnect though, complexity gradually aligned itself with sociology in a certain way: it enhanced our understanding that the ‘normal’ state in social settings is ‘not one of balance and repose’ but ‘recovering from the last disaster’ (Urry 2005, 6). For example, through its oeuvre, complexity elaborates that sociology is not as interested in equilibria (as e.g., economics is) but inherently interested in the tipping point, the emergent and the deviant (as economics is not; Page 2015). Indeed, complexity ‐due to its focus on unpredictability‐offered a vantage point to elucidate key sociological themes that seemed marginal and did so via a deeper layering that an epistemological monoculture alone could not offer (Urry 2010).

Thus, complexity elevated sociology's status within the sciences for two reasons: First, adjacent fields such as economics or psychology cast a narrower gaze at the transdisciplinary and wicked reality that a sociologist must attend to (Viskovatoff 2000). On the contrary, sociology ‐via complexity‐cemented its status as the study of multiple interactions in situ that is, contexts that play a crucial role in explaining outcomes (Page 2015). Second, sociologists are interested in the deviant and the unmarked (Scott 2018), as well as in situations that are prone to change and require action (Stehr and Grundmann 2001). Complexity became the theoretical arsenal that lent itself to both ‘deviant’ and ‘actionable’ inquiry since it is not preoccupied with systematicity, determinism and linearity but with non‐linearity and emergence. Instead of presuming equilibria or formalizing observations through invariant laws, a big part of sociology embraced complex situatedness to ‘explain’ structures and ‘illuminate’ agentic action (see Urry 2010).

This progress is exemplified in theorizing that transcends positivism/post‐positivism dichotomies (Gerrits 2012); in translating findings to resonate with certain audiences (e.g., via metaphors and analogies; Castellani and Gerrits 2024); or, in underscoring how social complexity, unlike natural settings, is driven by purposive actors (Room 2016). Such advances sum up to a social science turn in complexity (Castellani and Gerrits 2024), which moved complexity studies beyond: canonization of concepts as inherently relevant (e.g., scale‐free networks); borrowing from other fields (e.g., Pareto principles); glorification of methods (e.g., simulations) or assumptions (e.g., equilibria); and a priori predictive or a posteriori descriptive portrayals of complexity (see Byrne and Callaghan 2022; M. Williams 2021; Room 2016). Instead, the ‘turn’ emphasised: inclusive methodological toolkits (e.g., interviews and observations); accounting for the historical and open evolution of social systems; idiosyncratic agentic concerns (e.g., the struggle for positional advantage); and direct implications for practice.

Hence, the social science turn was not just about methods. Rather, it involved a deeper shift in how complexity is understood, particularly around the role of agency and the centrality of situated judgement. Due to this fundamental shift, the field has reached a level of maturity and still produces fascinating results. What is still missing though is missing‐ness; a focus that can further enrich these agentic developments via a parallel ‘missing’ turn in complexity.

## 4 Complexity in Sociology of Organizations

An area that epitomizes aforementioned remarks is the sociology of organizations (e.g., civil societies, voluntary communities, the military, social collectives, political parties, activist groups, non‐profits, multinational corporations, teams, supranational organizations, charities, or small firms). Such organizations and their constituent parts (people, departments, resources) are routinely portrayed as entities that interact with others (citizens, the state, colleagues, markets, institutions) to yield entitative outcomes (e.g., drive social change, generate profitability, maintain peace, enact shared meaning). Thus, an utter entitization in terms of structure, content, means, or purpose is inextricably intertwined with the field's development at the expense of what is missing (see Giovannoni and Quattrone 2018, 2025; Knox et al. 2015 for a requisite critique).

Exceptions that loosen ‘strict Weberian hierarchies’ and ‘scientific purity’ as the best ways of organizing social settings exist (Castellani and Gerrits 2024, 352, 354). For example, science and technology studies of invisible work in organisations or ignorance studies can enable practitioners or policymakers to deal with complexity challenges as real‐world problems (Room 2016). However, these are exactly that (exceptions) whereas several of those studies claim no link to complexity theories. Contrarily, in complexity studies per se, we routinely observe entitative terms such as:

– *‘proliferation’* of essentially self‐identical entities and their interactions (Piazzai and Wijnberg 2019) as opposed to a process of emptying out or lack;
– increased *‘variety’* (Boisot and McKelvey 2010) as opposed to absence and a disintegrating sparsity;
– heightening of *‘activity’* (Ganco et al. 2020) as opposed to non‐action/passivity;
– *‘accumulation’* and concentration of detectable states and salient events (Van Oorschot et al. 2013) as opposed to dispersion or hardly observable cues.

What is *not* evident then is the effect of missing elements on the behaviour of organisations as complex structures and on organisational actors who may feel unable to understand, confront or absorb the complexity that surrounds them. This lacuna comes as no surprise. e.g., organisations are often framed to emerge from the ground up and scale up to organized complexity. Not only does this framing ignore critical issues (e.g., power, racism, discrimination, sexism) but also focuses on interactions between the ‘plentiful’ and the ‘evident’. Subsequently, organisational sociologists often go on to do their craft without due consideration for omissions, lack, mortality, rupture and gaps (Giovannoni and Quattrone 2018, 2025; Crosina and Pratt 2019). However, such non‐states are instrumental in making things happen in and around organisations. In fact, a discerning outlook that allows organizational agents to be ‘reliably and efficiently informed about what is absent from the world’ has requisite effects on their ability to plan, predict, or act (Farennikova 2013, 435; see also Barton 2020).

Therefore, in organizational sociology, problematizing entitative assumptions is important for two reasons: First, organisations that act on the basis of *only presence‐laden cues* are exposed to a threat; or else, they risk decay and collapse. Alternatively, if we miss half of the complex story that we are trying to decipher, how credible are the findings of requisite scholarship? This is not a peripheral concern but substantially impacts the ability of organizational actors to anticipate, imagine, perceive, prepare, synthesize; or, simply, organize. Second, the word ‘complex’ is a ubiquitous adjective to describe properties of contexts that sociologists study (e.g., institutions, communities, states). Specifically, organizational sociologists strive to identify, measure and model those ‘complexities’ and respond through adaptive strategies that enable an organization's performance, the latter being the Holy Grail of their scholarly craft. Missing‐ness, given its ontological thinness, seems to be incongruent in this matching contingency and irrelevant in grasping complexity. Yet, it is ubiquitous leading to a serious scholarly disconnect.

## 5 Demonstrating Entitative Commitments

The typical notion of complexity in organizational sociology is that of a complex adaptive system, which refers to ‘a whole comprised of a large number of parts, each of which behaves according to some rule or force that relates it interactively to other parts’ (Maguire et al. 2006, 166). Then, complexity denotes the quantity (variety) and interactions (connectedness) between discrete and definable entities (Page 2015). That is, observable and bounded elements in a clearly specified spatio‐temporal setting interact non‐linearly with each other and, given sensitivity to initial conditions, these interactions give rise to grander outcomes (Urry 2005; Boisot and McKelvey 2010). Therefore, complexity scholarship (i) is evidently based on a quantitative understanding whereby *many interacting elements* define its nature as turbulent, fast‐paced, or dynamic (ii) largely focuses on how organizational structures emerge and self‐organize following interactions of agents at lower levels of analysis or through actual inputs from the external environment (Maguire et al. 2006; Chiles et al. 2004). Therein, some characteristics stand out:

### 5.1 Sensitivity to Initial Conditions

The term denotes something specific, locatable and identifiable in space‐time that can be grasped, sensed or observed through our perceptual abilities for example, a communal breakfast inducing radical change (Plowman et al. 2007) or a lake, a book and a train giving rise to an organizational collective (Chiles et al. 2004). This form of theorizing rests squarely on a metaphysics of substance that privileges ‘what is’ over ‘what is not’. It assumes that the world is made up of discrete and substantive elements that can be ‘said to be *here* in space and *here* in time, or *here* in space‐time, in a perfectly definite sense’ (Whitehead, 1926/1985: 62).

It is this presumption that the world is made up of a ‘succession of configurations of matter’ that has captivated thought since the Enlightenment.

We cannot wonder that science rested content with this assumption as to the fundamental elements of nature. The great forces of nature, such as gravitation, were entirely determined by the configurations of masses…this is the famous mechanistic theory of nature, which has reigned supreme ever since the seventeenth century.

(Whitehead, 1926/1985: 63)

But this ‘spatialization’ of things is a distortion of nature, a ubiquitous error of ‘mistaking the abstract for the concrete’ that philosophy ‐unlike the sciences‐is well‐versed in (see Whitehead's ‘fallacy of Misplaced Concreteness’: 64). Overall, the idea that initial conditions are singular events located in a social space‐time that can be straightforwardly ‘sensed’ and mobilized as the basis of complexity in social settings belies continued commitment to a Newtonian model of the world (Whitehead, 1926/1985: 58–59). It is a system of comprehension that privileges entities over what is missing.

### 5.2 Self‐Organized Emergence

Following this sensitivity to initial conditions, complex arrangements emerge and self‐organise; they amalgamate into an entitative meta‐being out of otherwise prior discrete and autonomous entities (actors, resources etc.). Yet, this self‐organized emergence adheres to an atomistic view of the universe, too. In social sciences, the carrying over of this form of thought takes the form of ‘methodological individualism’ in which prior existence of a ‘self‐contained individual confronting a world “out there”’ is assumed (Ingold 2002, 4).

Thus, we find claims that ‘[s]elf‐organized strategic activity denotes the creation of an emergent dynamic pattern from the interactions between strategic actions.’ (Thietart 2016, 774) and that organizational emergence ‘results from the interdependence of system components’ (M. Schneider and Somers 2006, 353). Such theses presuppose characteristics of organizational actors that are already bestowed upon them in advance of their interactions and that such interactions do not materially affect their internally specified nature. Interactions are thus carried out by discrete, bounded entities by means of ‘external contact that leaves their basic, internally specified nature unaffected’ (Ingold 2002, 3; see also ‘population ecology’ theories). Again, there is no room for missing‐ness in effecting complex outcomes.

### 5.3 Order

Order is the teleological cornerstone of emergence, which is deemed to ‘arise [s] from the aggregated behaviour of interdependent entities… driven by a set of rules’ (Thietart 2016, 776). The presumption is that order spontaneously emerges through autonomous entities interacting by following established rules. How the rules come to be remains unclear; do they derive from universal immutable laws? The presumption seems to be that rules themselves are fixed entities that make up some kind of ‘pre‐ordering’ impulse. There appears to be little attempt to address formation of rules themselves, which explains the resultant ‘emergence’ we observe and study in social settings.

Be that as it may, in their search for fit, organizational entities coevolve through these interdependencies and ‐after adapting to environmental cues (competitors, technologies, customers; Van de Ven et al. 2013)‐ order is thereby attained (Osborn and Hunt 2007). Thus, tangled interdependencies between distinct entities feature as key across complexity traditions. Any ‘critical’ look upon them neither attends to nor does it challenge their ‘entitisation’. Rather, it refers only to their variation for example dense or weak ties that enable integration or differentiation; rigidity or learning (Ganco et al. 2020; M. Schneider and Somers 2006).

### 5.4 Adaptation

Order, emergence and self‐organization –as system‐level by‐products of complex interactions‐are brought about through adaptation by distinct and autonomous entities (Page 2015; Boisot and McKelvey 2010). ‘[P]rinciples (rules) of local interaction… require each agent to adjust its behaviour to that of other agents’ (Burnes 2005, 78–79) and this co‐evolutionary process forms the resultant landscape, which ranges from ‘small’ accomplishments such as team learning outcomes (Vashdi et al. 2013) up to the formation of whole meta‐structures such as rugged ecosystems (Ganco et al. 2020). Therefore, adaptation is the typical act in complex regimes so that it constitutes ‘the underpinning nomological network of the entire field’ (Poulis and Poulis 2016, 518). In fact, that's how social reality is shaped according to complexity theorists; via *co‐evolution of mutually adaptive, interdependent entities* (see Page 2015). As such, the situated specificities of each ‘miniscule’ becoming (other than adaptation) remain rather uncharted (Child and Rodrigues 2011).

### 5.5 Summary of Entitative Commitments

Entities then dominate the organisational discourse. Accounting for *known* forces, *current* environmental cues, *self‐evident* constraints and *‘seen’* adjacent entities is achieved ‘by searching a space (i.e., landscape) that has a set of predefined characteristics’ (Ganco et al. 2020, 652). Namely, the assumption is that social collectives respond through adaptivity with complexity theorists predominantly focussing on the entitative outcome (systemic order), the salient means (adaptive decisions) and the self‐emergent process of ‘becoming’ that mediates and links the two (Chiles et al. 2004). These ‘present’ manifestations (both present‐as‐contemporary and present‐as‐visible) are what complexity scholars routinely prioritize: they isolate fixed, recognizable entities resulting in entitative meta‐structures (M. Schneider and Somers 2006; Weigelt and Sarkar 2012).

Then, the complexity literature is predicated on notions of presence and being whereas missing instances such as lack, non‐action or omissions are curiously absent. Resulting from this assumption base, *system‐level properties* of social structures (their emergence, self‐organization, order) are complexity scholars' core orientation. Contrarily, a symmetrical focus on the underbelly propping up such visible structures is rare. For example, how such entities are delivered to our attention through the gradual marginalization and extraction of figure from ground is nowhere to be found. Moreover, serendipitous and accidental integration of entities, *pudenda origin*, into a surprisingly coherent complex whole remains uncontemplated (Chia 2011); the cautious unorderly arrangements within organizations involving the gamut of prescience, intentionality, foresight, prudence are left out of complexity analyses (Greenwood and Suddaby 2006; Poulis and Poulis 2016). In other words, agentic acts, or seemingly inconsequential non‐action and omissions, that go beyond the emergent, the organized, the adaptive and the ordered, do not feature as prominently in complexity accounts. In fact, such non‐adaptive stances are considered irrational (see Child and Rodrigues 2011).

## 6 Proposing a Complementary Treatment

Critical voices exist. e.g., some theorists do not understand complexity as the by‐product of a state of equilibrium (e.g., see focus on order; Osborn and Hunt 2007) or the quest for optimality (e.g., see performance maxima in fitness landscapes; Ganco et al. 2020), but in terms of becoming. This understanding is linked to process philosophy and, with it, notions of ceaseless change and transformation. Yet, even then, the goal is identifying universal laws that govern complex systems as a whole (A. Schneider et al. 2017). The focus remains on the present state of complex collectives; of how complexity becomes and how those structures have evolved. Curiously, there is no concomitant focus on what they have never been, are not or may never become. By and large, even those who purportedly embrace process thinking, construe process as epiphenomenal to entities and stable states; they remain trapped in a metaphysics, which privileges actual entities over missing‐ness. Hence, I appropriate missing‐ness as a fecund basis for grasping complexity along three fronts.

### 6.1 The Ontological Treatment of Complexity: An Additive Being Versus The Big Unknown

Aforementioned points reveal a paradox. On the one hand, there is consensus that complexity implies action on the basis of ignorance, randomness and uncertainty since social actors are largely agnostic of a system's properties and orientation (Page 2015; A. Schneider et al. 2017). As such, one would expect that the missing, the unknown, the unobserved –as the essence of complexity‐would systematically feature in analytical accounts. On the other hand, though, as shown, complexity is reified and understood on the basis of what ‘there is’ and ‘what is observable’; not on what there ‘isn't’ or is ‘unobserved/able’. As such, our engagement with complexity is rarely portrayed as *managing the non‐knowledge associated with its nature*. Rather, the portrayal focuses on (i) lower‐level interactions or, (ii) at the system‐level, description of general properties such as for example, the recursive symmetry, the pace of change or the scale dependence within the social system (Urry 2005; Page 2015).

By downgrading an agentic perspective though, we ignore something critical: missing‐ness is what often makes a complex regime appear as complex per se. If we were knowledgeable or could readily observe missing forces, then complexity itself would disappear, or it might turn out to be manageable. In essence, it is our epistemological limitations in accounting for unobserved forces of reality, which create this ontological truism: Complexity theorists depict complexity as a property‐laden structure; as the interactive configuration and amalgamation of entities into a contingent meta‐Being *in an additive sense*. In turn, actors are portrayed as knowledgeable agents who orchestrate adaptive responses that make up this meta‐Being.

Thus, an inevitable knowledge void (i.e., the real complexity that actors face) is surreptitiously replaced by a reified structure called ‘complexity’. This accumulative*, void‐filling* logic reflects how the agentic management of complexity is represented in requisite literature. Instead, a counterintuitive, *void‐reflective* logic that may deal with missing elements of complexity is largely absent; this, despite the recognition that ‘organizing’ is about skillfully navigating this open‐endedness (Chia 2011).

This framing of complexity as an additive being then renders scholars unable to suggest an appropriation of complexity as ‘the big unknown’. Subsequently, we nurture social actants (e.g., policymakers) who are not as equipped to exercise phronesis and intuitive judgement linked to complexity. For example, fixation with ‘knowledge economies’ has made us less attuned to the uncertainty associated with complex challenges (Davies 2011). Specifically, policymakers need to project unassailable epistemic authority since allocation of public resources is involved, and policies shall depend on the soundness of what policy experts suggest. Hence, recommendations are based on illusions of knowing and expertise, not on a parallel sensibility of not‐knowing, which is seen as a weakness.

Subsequently, we pretend as if tacit dealings with missing aspects are not happening ‘out there’. Yet, such dealings exemplify the routine management of complexity by social actors and make complexity challenging enough. Hence, while complexity is often understood as a grotesque entity or as one of gargantuan proportions, options to mitigate, overcome or confront the big unknown such as for example, doing ‘nothing’ against complexity or ‘not becoming’ by ignoring it are far less common (Poulis and Poulis 2016). Rather, as noted, the scholarly effort is to measure this additive Being and do something *against* it, which usually takes the form of adaptive matching (Boisot and McKelvey 2010).

Yet, even in cases where complexity is recognised as the big unknown (e.g., complexity absorption strategies; Maguire et al. 2006), epistemological choices therein are misaligned with this ontological outlook. Specifically, a rush into quantification, co‐variance and reductionism –in line with a Newtonian ideal‐is utilized by classifying, coding, and linking abstractions of, always, entitative manifestations of complexity (Weigelt and Sarkar 2012). This leads to epistemology.

### 6.2 The Epistemological Treatment of Complexity: Measurement Versus Articulation

Complexity scholars routinely appropriate simulations, network clustering algorithms, differential equations and variance tools to measure (e.g., Kauffman’s (1995) NKC model) or model (e.g., law of requisite variety; Boisot and McKelvey 2010) complexity arrangements. Mathematical formalism and a quest to attain numerical precision are characteristic of this tendency of additive reification for example, by measuring structural variety (N) and its internal (K) and external (C) inter‐connectedness (Van de Ven et al. 2013). Narratives, articulation, illumination of complexity are seen as sub‐optimal modes while measurement seems to be the proper tool applauded by our epistemological conventions (see Cannon and John 2007).

In this way, we miss opportunities to elucidate the ontology highlighted before (i.e., complexity as the ‘big unknown’) and make it relevant for theorizing. What can we recommend to social actants or policymakers when complex forces are unobserved and hence, unmeasurable? To such a question, we pretend that there is no available answer only because the measuring legacy rooted in our epistemological priorities cannot appropriate missing‐ness. The net result is learning tools that promote statistical mastery but not situational awareness and judgement. This is mostly evident in the use of simulations, which seem as if they capture fragments of complexity through a mystic quality. However, by simulating reality, we refrain from understanding more elusive dimensions. Then, how can we expect social actors (the endpoints of our epistemological attention) to address this ‘big unknown’ via our learning tools?

Example Zhao and Ge (2023) explain how institutions oscillate between isomorphic tendencies and differentiation and, from an agency perspective, Clark (2021) shows how victims of violence navigate adversity in conflict‐stricken contexts. Yet, to demonstrate institutional integration or how victims build resilience, emphasis is largely on present systems and environmental cues (e.g., due to the frictions and disturbances that those entail). In line with a complexity nomenclature, studied participants navigate contexts ‘*in connection with* other elements (i.e., sub‐systems) within their social ecologies’ (Clark 2021, 1050; emphasis in original). Then, several studies embrace process and flow but, still, there is an entitative emphasis on nodes, actors, roles, positions and their interconnections. In fact, ‘[i]n the absence of interaction and concrete relations between social actors there would be no society and no structure to talk about’ (Crossley 2022, 179). Yet, often, it is because of such absences that new social arrangements emerge. For example, silence in the workplace diminishes employees' creativity (Morrison 2023) and parental loss redefines our presence in the world (Poulis 2025). So, aspects of missing‐ness such as its effectuality (e.g., negative or positive) or its temporality (e.g., entities that used to be or ones that may become) routinely enact new social structures.

### 6.3 The Analytical Treatment of Complexity: The Measurable Versus The Unknown

Given these commitments, scholars understand complexity as a series of measurable *constraints*. Against the latter, adaptive ‘fitting’ appears as the only available response (Weigelt and Sarkar 2012; Boisot and McKelvey 2010). Cognitive rationality and calculative intentionality towards *adaptation to a co‐evolving ‘being’* thus appear as the desiderata of complexity management. Simply, in complex regimes, ‘proper’ organising is equated with adaptivity while non‐adapting is portrayed as irrational and disastrous (Uhl‐Bien et al. 2007; Child and Rodrigues 2011).

However, dealing with complexity is not only about adapting to a neighbouring entity's moves nor are complexity elements merely constraints. Rather, a hidden and ineffable ‘otherness’ is also responsible for the catalytic creation of meta‐structures. It is the inconclusive polysemy of the ‘big unknown’ that *ipso facto* makes complexity a scholarly pursuit. This non‐knowledge may seem daunting and unwieldy to manage but it is the social reality we investigate and participate in. Yet, there is no *explicit* treatment of missing‐ness. Rather, ‘actual’ entities or ‘entitised’ relationships are at the foreground of inquiry whereas the a‐relational, the absent or the enigmatic are treated as unimportant.

I ought to clarify: Both ‘entitative’ and ‘process’ exceptions exist on risk (Deville and Guggenheim 2018), uncertainty (Hansen and Borch 2021), unknowns (Giovannoni and Quattrone 2025), disasters (Collier 2025), or absence (Poulis 2025), which indicate liminal spaces of potentiality and worlds in the making. Moreover, studies acknowledge something i.e. no more (e.g., memory as antecedent of movement; Clark 2021) or something i.e. yet to become (e.g., potentiality as a by‐product of ‘flows’; Lee 2024). So, my critique is not about a general lack of sociological interest in the absent, the non‐actualised, the non‐knowledge, or the unmarked. Rather, my critique is limited to complexity traditions in sociology, which are reluctant of embracing missing‐ness.

This is important because social actors are not effortless processing machines of only observable forces of reality. Rather, dealing with complexity takes place amid *epistemic incompleteness*. This imperfect state implies that our analytical foci (professionals, community leaders, employees, activists, craft makers, citizens) must skilfully navigate the missing and the ghostly in a certain agentic way: It is not only their knowledge of being‐laden complexity that makes their action possible, meaningful or desirable. It is not only contingently configured *beings* that guide, orient, direct or inspire their choices. Missing‐ness matters, too since it leads to fundamental uncertainty (Pixley 2002). After all, any ‘actual’ knowledge of reality that sociologists try to capture is sub‐optimal because of complexity's colossal nature. Thus, relying on ‘actual knowledge’ to deal with complexity is not only ontologically myopic; praxeologically, it is a sort of hubris, too.

For example, the sociology of ignorance (Mueller 2018; McGoey 2012) frames not‐knowing as a generative fact, not an impediment. It catalyses indifference, racism, exclusion and, thus, produces social effects. From a positive perspective, it energizes actors to visualize previously unimagined possibilities and search for new solutions (Kier and McMullen 2018); or materialise own choices via tacit doings and learning (Poulis and Poulis 2016). Then, missing‐ness induces and enacts; it creates potentialities and pinpoints what is possible (e.g., activists refusing to accept lack, gaps, or omissions). Social action then is not born only out of what is known but rather *due to* what is unknown or yet unseen. This missing‐laden rationality is what agents are expected to deploy in complex settings, that is, to demonstrate prudence amid what they neither know nor possess. Table 1 grasps those nuances by summarizing existing (left) and proposing a complementary treatment (right) of complexity.

**TABLE 1:** The extant treatment of complexity and a complementary proposal.

Extant treatment of complexity	A proposal
Ontological level	
An entitative being (an entity of gargantuan proportions)	The big unknown (non‐knowledge of missing imperatives)

Epistemological level	
Mathematical formalism and nomothetic regularities Effort to tame and measure complexity as an additive meta‐Being Excessive use of simulations, network clustering algorithms, differential equations, variance models Effort to attain numerical precision for matching purposes Predictive and generalizable orientations as epistemological desiderata	Narrative and articulation Probabilistic inference to surmise empirical tendencies Possibilities to illuminate non‐typical acts Account for ‘invisible’ forces aggravating complexity Focus on what we never were, may lose or may never become

Analytical Level	
Entities framed as measurable *constraints* Resulting to doing ‘some‐thing’ *against* them Portrayed in the form of *adaptive matching*	Previously unimagined possibilities Preemptive‐ness in relation to empirical tendencies Reproduce or enact own choices Doing nothing by disregarding complexity

## 7 Discussion

According to theorizing orthodoxy, truth ‐as the teleological cornerstone of science‐is accessed through rigorous observation, faithful documentation and the subsequent development of tight causal explanations. It cannot be an incidental by‐product found in the idiosyncrasies of multiple tacit doings, or in inherent ambiguities associated with an infinite discourse. Instead, truth must be based on what our senses, relying on well‐established and rigorous protocols, are able to observe, analyse and report as properly verifiable knowledge. In organizational sociology, this prevailing ontological outlook implies certain epistemological commitments that allow ‘building causal models that seek to optimize some form of organizational success’ (Grewatsch et al. 2023, 721).

Truth‐finding then is an entitative pursuit; attained via the dispassionate collecting of ‘facts’, the measurement of ‘data’, the analysis of ‘evidence’ and their linking together in a comprehensive framework of causal relations. This is not unique to organizational sociology; such neo‐Parmenidean reification of concrete entities as the basis of reality is evident across many fields and provides the means for reaching truth via strict adherence to established protocols of scientific rationality. Why is this rationality problematic though? Largely, because of a ‘cognitive access problem’ that is, missing instances set an impenetrable barrier to complexity theorists who must somehow appropriate such instances in their accounts (Barton 2020). But how can scholars identify and report something so ontologically thin as ‘missing‐ness’? Ultimately, missing elements are inconclusively circumscribed for analytical purposes, and, given our fundamental empiricism, entities take centre stage. As a result, complexity scholars ‐despite the promise to elucidate a wholesome reality‐are early entrained to appropriate ‘what is’ rather than ‘what is not’.

These substantialist assumptions, though logical, remain problematic. Namely, they downplay invisible antecedents at play and do not countenance as‐yet non‐existent possibilities. They engender a comforting epistemological stance for scholars: By treating complexity as an additive set of selected abstractions (variables) that are *‘defined, structured and predictable’* (Vashdi et al. 2013, 951) and linked through certain rules, complexity can be measured and thus, ‘tamed’ and controlled despite its unwieldy nature. Without this tendency to reify entities, measurement of complexity would have been impossible; Aristotelian empiricism would be threatened, and Newtonian tools would be less helpful. Then, we witness a paradox: Instead of ontology dictating epistemological means to capture complexity, our substance‐laden epistemological tools negated an ontological aspect related to complexity—that of missing‐ness. This reverse order is a scholarly distortion, a fundamental theorising flaw. The role of epistemology is not to decide *what* is to be known; this is the territory of ontology. Yet, epistemology—as the mode of knowing that seems, however, unable or unwilling to grasp what is unobserved—has limited our ontological scope to *actual and observable presences alone* in articulating complexity.

For example, in parts of social sciences, ‘missing‐ness’ is, at best, used as a counterfactual example or a passing reference; had *a* occurred, *a* would have caused *b* (Dowe 2009). However, in sociology, non‐occurrences, inaction, omissions, gaps can for example prevent, inspire, impede, orient or enable. Namely, as noted, missing circumstances play *a functional role* (Tiehen 2015). Despite this effectuality though, we are almost instinctively attracted to the manifest, the visible, and the eye‐catching, a legacy of Aristotle's insistence in *Metaphysics* that ‘[o]f all the senses, sight best helps us to know things, and reveals many distinctions’ (qtd. in Kambaskovic‐Sawers and Wolfe 2014, 110). The cultivated tendency, as such, is to focus on the immediately visible in explaining social phenomena and in formulating action imperatives and, thus, neglecting or not attending to that which is implied as missing or that which remains unobserved/unobservable (Chia 2011; Scott 2018). By extension, an overriding tendency in the complexity discourse, too is to focus on pure manifest, well‐defined forms—to ‘the palpable, the real, the visible, the concrete, the known, the seen, the vivid, the visual’ (Taleb 2007, 132)—and to overlook less‐defined structures and processes. Less observable aspects are nebulous, and their dubious reification does not conform to the being‐laden empiricism of complexity accounts.

Contrary to conventional wisdom, this study raised a heretic point: Complexity in sociology is broadly understood as entities (actors, objects etc.) that interact via adaptive responses implemented by victims of violence, professionals, athletes, immigrants, social workers, or criminals (Clark 2021; Page 2015; Roberts et al. 2017). However, why should an event *occur* or actors *interact* before we theorize underlying processes constituting complexity? Can't complexity be constituted through e.g., a lasting absence? Such lack may be proven unwieldy and due care for it might prevent detrimental effects linked to chaotic regimes (e.g., via effects of radical change and unpredictability). Yet, such complexity framing is virtually inexistent. Then, this paper urges complexity scholars to consider two things:

- First, a vulnerability thesis that is prominent in sociology. Namely, social reality is ephemeral, mutating and fleeting; or else, inherently infused with a missing potential. Thus, despite the omnipresence of complex ‘beings’ in our accounts, one ought to revisit the premise that social complexity is all about ‘entities’. Otherwise, we miss opportunities to elucidate how missing‐ness induces complex regimes.
- Second, a complexity mantra, which is nominally recognized but ‘desecrated’ in scholarly practice: Complexity is not an a priori, objective property of a system; it is the *a posteriori*, subjective property of that system, which is contingent on our perceptual and interpretation powers. Given this phenomenological property, our units of analyses must be agents, not variables (Page 2015).

Both remarks imply that complexity cannot be subject to predictive generalizations. For example, there are no universal truths that allow us to foresee complexity since behavioural inclinations or cultural drives at the micro‐level matter. Rather, we can surmise macro‐level empirical tendencies, localized solutions and probabilistic approximations or a posteriori record accomplished structures. This leads to *a disconnect*: On the one hand, sociology does a superb job in investigating unachieved social goals that imbue a sense of loss (Aarseth 2018); the image of a looming missing‐ness (e.g., death) as a possibility that ignites dread (Walter 2012); voids that may lead to preservation of norms (Gezelius 2007). On the other hand, complexity scholarship has not followed suit. At best, it records randomness in agentic choices or quasi‐permanent equilibria of structural configurations.

Attunement to missing‐ness though is *not just* about describing a new kind of complexity. Rather, such complexity has important implications *for social action*. Namely, this study recognizes a functional role for missing instances; they are neither ghostly instances nor distant possibilities. Rather, social actants and scholars alike must *cope with* these missing objects, persons, or circumstances. e.g., emergence ‐a principal complexity concept‐implies some kind of causal agency in social settings where ‘things’ do not emerge automatically. By ignoring missing‐ness then, we pretend as if its manifestations (e.g., absence, loss) do not impact upon actors. Yet, as noted, missing‐ness implicates with our presence in the world; it intensifies our ontological insecurity, alerts us that something is wrong, or galvanizes corrective action (Poulis 2025). Then, how can scholarly accounts explain complex outcomes if they neglect something so fundamental for human agency as missing‐ness?

This perspective may seem unnecessary if we seek to *describe* established realities. However, in social sciences ‐where ‘the social’ is not viewed only macroscopically but also studied in grounded terms‐this agency is largely its raison d'être. Alternatively put, complexity scientists do a fine job in modelling accomplished structures but shed less light on the accomplishers (their anxieties, fears, perceptions, biases etc). e.g., missing resources leads to despair and social actants (e.g., citizens in underprivileged communities) may interpret this lack as a calling. Moreover, a projected network exit may enable community leaders (e.g., social activists) to extend their presence in another network or stubbornly preserve it to impede mutation to an undesirable self. Then, missing‐ness enables a deep caring for one's identity and a concomitant attempt to change, restore, refine or preserve existing practices (Poulis 2025; Scott 2018).

Scenarios vary and imply situations when the existence, reliability or identity of a nation, an organization, or a community may be at stake. In a nutshell, missing instances underscore agents' attempt to safeguard what matters. Missing‐laden complexity then is not neutral. Rather, it may propel agents to act; an agency that would otherwise remain dormant or not thought‐of. Then, by focussing on ‘was not’, ‘is not’ or what ‘may be missing’, I call for actionable possibilities that remain empirically undocumented. What is suppressed or overlooked by this dominant metaphysical orientation towards entities has consequences in terms of what we know, how we act and the subsequent outcomes that ensue. We neglect to elucidate the effectuality of an event that never happened, an occurrence that will not materialize, cues that are missing yet projected that is, ‘unseen’ instances that beg for illumination of their complexifying potential.

## 8 Conclusion

The complexity we try to disentangle is not only what we immediately capture through our perceptual abilities. Decoding social complexity often resides in the ineffable, the unarticulated, the un‐thought of and the mundane; all being linked with manifestations of missing‐ness. Then, a missing turn on reality's ‘invisibles’ can rejuvenate the social complexity literature, which is marred by several challenges: few or inconclusive attempts to philosophise, single levels of analysis, or an overly technical nature at the expense of theory (Castellani and Gerrits 2024). In fact, a new missing‐laden imagination coupled with interdisciplinary insights (see Yang 2024; M. Williams 2021) can be a meaningful response to Morin's (2007: 23) plea for elucidating a key concern: ‘What is complexity’? Yet, this imagination is not to imply the need for a clean historical break from extant traditions. Rather, a missing turn is envisaged to develop through overlapping or coexisting strands, which already offer exceptional insights. Thus, this paper must be seen as a gesture towards dialogue with adjacent traditions, rather than a new turn as such. Otherwise, any radical embracement of missing‐ness and a parallel ignoring of entitative traditions would oppose the plural and open‐ended history of complexity that this paper tried to describe.

## Funding

The author has nothing to report.

## Conflicts of Interest

The author declares no conflicts of interest.

## Data Availability

Data sharing not applicable to this article as no datasets were generated or analysed during the current study.
